# Salivary IL-1β, IL-6, and IL-10 Are Key Biomarkers of Periodontitis Severity

**DOI:** 10.3390/ijms25158401

**Published:** 2024-08-01

**Authors:** Marta Relvas, Ana Mendes-Frias, Maria Gonçalves, Filomena Salazar, Paula López-Jarana, Ricardo Silvestre, Alexandra Viana da Costa

**Affiliations:** 1 University Institute of Health Sciences (IUCS-CESPU), 4585-116 Gandra, Portugal; mprazeres.goncalves@iucs.cespu.pt (M.G.); filomena.salazar@iucs.cespu.pt (F.S.); paula.jarana@iucs.cespu.pt (P.L.-J.); alexandra.costa@iucs.cespu.pt (A.V.d.C.); 2 Oral Pathology and Rehabilitation Research Unit (UNIPRO), University Institute of Health Sciences (IUCS-CESPU), CRL, 4585-116 Gandra, Portugal; 3 Life and Health Sciences Research Institute (ICVS), School of Medicine, University of Minho, 4710-057 Braga, Portugal; id8209@alunos.uminho.pt (A.M.-F.); ricardosilvestre@med.uminho.pt (R.S.); 4 ICVS/3B’s—PT Government Associate Laboratory, 4710-057 Braga, Portugal; 5 UCIBIO—Applied Molecular Biosciences Unit, Toxicologic Pathology Research Laboratory, University Institute of Health Sciences (1H-TOXRUN, IUCS-CESPU), 4585-116 Gandra, Portugal

**Keywords:** periodontitis, saliva, inflammatory cytokines, biomarkers, severity and progression

## Abstract

To explore severity and progression biomarkers, we examined the clinical relevance of multiple cytokines and mediators involved in the inflammatory response in periodontitis. A cohort of 68 patients was enrolled in the study and periodontal status assessed by the current classification of periodontal diseases. Immune mediators present in saliva, of both patients and healthy controls, were quantified using a Legendplex-13 panel. Clinic parameters were significantly higher in PD patients compared with HC, with a strong significant association with the disease severity (stage) (*p* < 0.001), but not with progression (grade). The panel of immune mediators evidenced elevated levels of pro-inflammatory cytokines IL-6 and IL-1β as disease established (*p* < 0.01). IL-1β/IL-1RA ratio was increased in PD patients, being associated with disease stage. An anti-inflammatory response was spotted by higher IL-10. Lower levels of IL-23 and IP-10 were associated with disease severity. No significant statistical differences were found by grade classification. Moreover, salivary IL-1β and IL-6 exhibited significant positive correlations with several clinical measurements (PI, BOP, PPD, CAL), while IP-10 showed a statistical negative correlation with BOP, PPD, and CAL. These insights highlight the complexity of the periodontitis inflammatory network and the potential of cytokines as biomarkers for refined diagnostic and therapeutic strategies.

## 1. Introduction

Periodontitis (PD) is a chronic inflammatory condition, presenting an impact on the alveolar processes, gingiva, dental cementum, periodontal ligament, and other tooth-supporting tissues [1]. PD develops due to a complex interaction of genetic, environmental, and biofilm factors, where dysbiosis of the oral microbiome triggers the initial inflammatory process that deepens into the gingival sulcus, forming a periodontal pocket [2]. The initial inflammatory phase is characterized by local gingivitis, which can be reversed to a healthy state with proper oral hygiene. If left untreated, it can progress to periodontitis, wherein the inflammatory immune response spreads to other periodontal tissues, such as the periodontal ligament and alveolar bone, resulting in an irreversible process. The increase of probing depth (PPD), plaque index (PI), bleeding on probing (BOP), and the reduction of the clinical attachment level (CAL), along with subsequent bone loss, characterize the severity and progression of PD [3,4]. While these classical parameters continue to establish the basis of clinical diagnosis, they do not provide reliable information on the current activity of the disease and its future progression. They also are time-consuming, error-prone, and not well tolerated by patients. The scientific community has a particular interest in finding quantifiable biomarkers in oral fluids that can improve early detection rates of periodontitis and evaluations of its severity and progression [5,6,7].

Saliva is a non-invasive easy-to-collect fluid, with minimal discomfort to the patient and reflects the inflammatory status of the whole mouth and is widely used by research teams, representing the location and presence of potential PD biomarkers [8].

Cytokines are inflammatory mediators believed to play a role in the transition from reversible gingivitis to irreversible periodontitis [2,9]. Notably, interleukin IL-1β, IL-6, tumor necrosis factor-alfa (TNF-α), and receptor activator of nuclear factor kappa-B ligand (RANK-L) have been shown to be pivotal in the pathophysiology of PD [9]. IL-1β was one of the first cytokines suggested as a relevant biomarker for periodontal disease [10,11] and has shown promise in early periodontal diagnosis using saliva [5,11,12]. Our team’s earlier study revealed a possible cooperative role for the pro-inflammatory cytokine IL-1β and the immune mediator RANK-L in inflammatory and bone loss events, by linking salivary levels to both stage III/IV and grade C of PD. RANK-L may function as a combined diagnostic biomarker for PD in combination with IL-1β [13].

Despite advances, it is difficult to find a consensus on the best biomarker for periodontitis, even based on a highly representative number of studies. Moreover, a better understanding of the inflammatory process in PD, considering the disease stage and grade, is crucial for comprehending the network of inflammation and, consequently, for identifying better biomarkers for an early and reliable diagnosis. To this end, our target was to evaluate a wide array of immunomediators (cytokines and chemokines) associated with the regulatory inflammatory response in PD patients, classified according to the consensus of the AAP/EFP [3], aiming to elucidate some aspects of the inflammatory events in PD.

## 2. Results

### 2.1. Demographics Characteristics and Periodontal Status

Sixty-eight participants were selected for this study based on inclusion and exclusion criteria. Among the examined ones, 22 were healthy controls (HC), 17 were patients with periodontitis stage I/II, and 29 were patients with periodontitis stage III/IV. Table 1 displays the population’s demographic characteristics. Although this condition is more common in male [14], no association was found between gender—47 female (69%) compared to 21 male (31%)—and disease stage (χ^2^ = 5.33, *p* = 0.070). To assess the impact of age on PD stage, three age groups were established to simplify the analysis: less than 25 years, between 26 and 45, and more than 46 years, following the literature [15]. We observed a statistically significant relationship between age and periodontal status (χ^2^ (4) = 28.75; *p* < 0.001), with a predominance of the disease in older subjects regardless of the disease stage. Among the 36 individuals aged over 46 years, 20 (55.6%) had periodontitis III/IV, while 13 (36.1%) had periodontitis I/II stages. Conversely, among the 11 individuals aged 25 years or younger, only one had periodontitis I/II stage. In relation to the behavioral variables that could potentially alter or worsen the illness, we found that 46 people did not smoke (67.6%), eight people had previously smoked (11.8%), and only 14 people smoked (20.6%). A statistically significant correlation was found (χ^2^ (4) = 9.54; *p* = 0.049) between smoking behavior and periodontal health. This association was not linked to the quantity of tobacco consumed, in any of the studied groups, given that no statistical relationship was found between the number of cigarettes per day.

**Figure 1:**
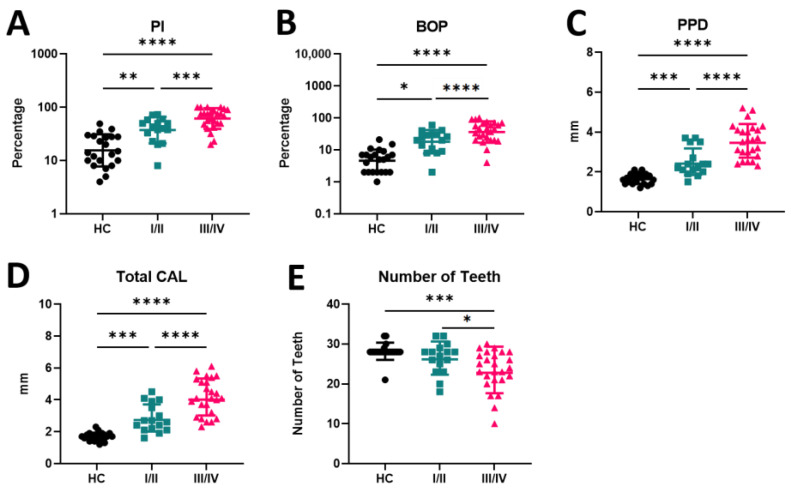
(**A**–**E**) Periodontal indices in periodontally healthy and periodontitis stages I/II and III/IV patients. PI—plaque index; BOP—bleeding on probing; PPD—pocket depth; CAL—clinical attachment loss; HC—healthy control; I/II—patients in stage I/II of PD; III/IV patients in stage III/IV of PD. * *p* < 0.05; ** *p* < 0.01; *** *p* < 0.00 1; **** *p* < 0.0001.

The clinical characterization of PD includes several measurements indicating disease development. The most relevant determinations are PI, BOP, PPD, CAL, and the number of teeth. PD patients in stage III/IV exhibit a significant increase in the levels of PI, BOP, PPD, and CAL compared to HC (*p* < 0.001 for all parameters) (Figure 1A–D). Also, patients in stage I/II presented a significant increase of these parameters compared to HC (*p* = 0.003 to PI; *p* = 0.026 to BOP; *p* = 0.008 to PPD, and *p* = 0.001 to CAL). Furthermore, there was a significant increase in all parameters in stage III/IV patients when compared to those in stage I/II (*p* = 0.001 to PI; *p* < 0.001 to BOP, PPD, and CAL) (Figure 1A–D). Conversely, the number of teeth significantly decreases with PD (*p* < 0.001), with patients in stage III/IV having significantly fewer teeth compared to patients in stage I/II (*p* < 0.001) and the HC group (*p* = 0.041) (Figure 1E).

**Figure 2:**
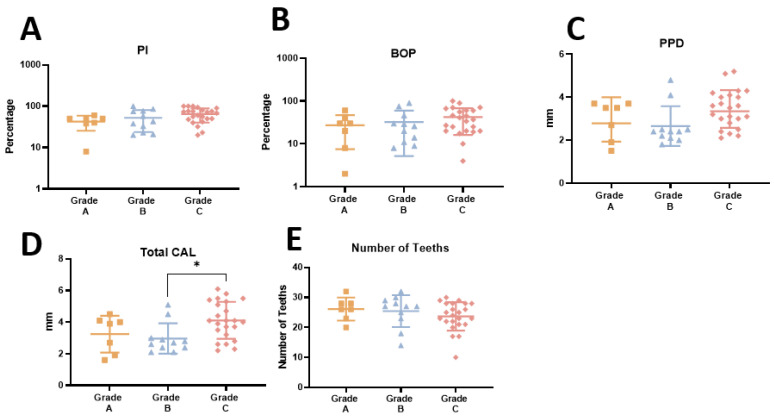
(**A**–**E**) Periodontal parameters in patients with grade A, B, and C of periodontitis. PI—plaque index; BOP—bleeding on probing; PPD—pocket depth; CAL—clinical attachment loss. * *p* < 0.05.

Among the 46 patients with periodontitis, 7 (15.2%) were classified as grade A, 13 (28.2%) as grade B, and 26 (56.6%) as grade C based on their rate of periodontitis progression, responsiveness to standard therapy, and potential impact on systemic health. When comparing patients according to disease grade (A, B, C), no significant differences were observed, except in the levels of total CAL of patients in Grade C that were significantly higher than in grade B (*p* = 0.012). (Figure 2D). It is worth mentioning that periodontal parameters tended to increase from patients with grade A to grade C (PI, BOP), with the opposite being observed with the number of teeth (Figure 2E).

### 2.2. Salivary Cytokine Discriminates Periodontal Disease Stages but Not Grades

**Figure 3:**
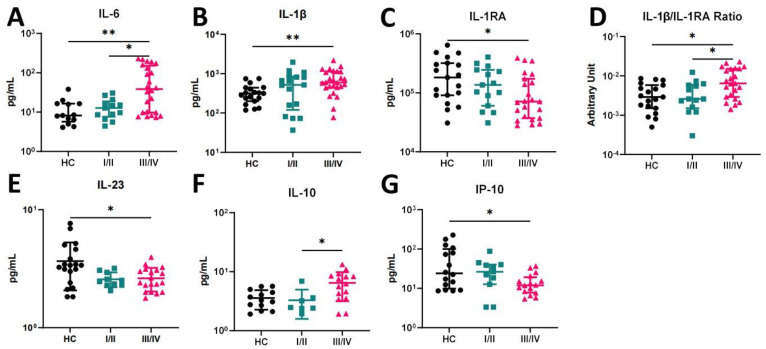
(**A**–**G**) Salivary cytokine profile in healthy controls and periodontitis I/II and III/IV patients. HC—healthy control; I/II—patients in stage I/II of PD; III/IV—patients in stage III/IV of PD. * *p* < 0.05; ** *p* < 0.01.

Saliva samples were collected for cytokine quantification from PD patients and HC. A pro-inflammatory profile was observed in patients with severe disease (stage III/IV), characterized by elevated levels of IL-6 and IL-1β compared to HC (*p* = 0.003 and *p* = 0.006, respectively) (Figure 3A,B). Moreover, higher levels of IL-6 were noted in patients in stage III/IV compared to those in stage I/II (*p* = 0.048) (Figure 3A). The increase in IL-1β in stage III/IV was accompanied by a decrease in IL-1RA throughout the disease, with a significant difference compared with HC (Figure 3C). Combining these two markers, a significant increase in the IL-1β/IL-1RA ratio was observed (*p* = 0.004), characterized by higher levels in stage III/IV patients compared to stage I/II patients and HC (*p* = 0.046 and *p* = 0.015, respectively) (Figure 3D).

Other pro-inflammatory cytokine levels were evaluated, including TNF-α and IFN-γ; however, no significant differences were found amonggroups. Interestingly, a significant decrease in IL-23 was observed in patients classified as stage III/IV compared to HC (*p* = 0.038) (Figure 3E). Regarding salivary IL-10 levels, a significant increase was noticed in stage III/IV patients compared to stage I/II (*p* = 0.047) (Figure 3F). The chemokine IP-10 was also quantified in the saliva of PD patients, as it plays an important role in orchestrating a proper inflammatory response in PD. Patients in stage III/IV exhibited a significant decrease in the levels of this chemokine compared to the HC (*p* = 0.045) (Figure 3G).

**Figure 4:**
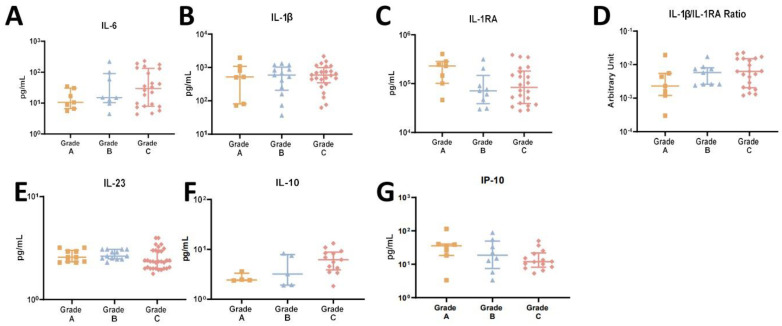
(**A**–**G**) Salivary cytokine profile in patients with periodontitis grade A, B, and C.

Following the clinical parameter approach, the cytokine profile across disease grades was assessed. However, no significant differences were found among disease grades (Figure 4A–G). Although no significant differences were observed among groups, we could identify a tendency in IL-6 levels to increase across grades (C > B > A). The same tendency is observed in IL-1beta/IIL1RA ratio and in IL-10 levels. IL-1β did not present a specific profile in grade. In opposition, IP-10 revealed a decreasing level from grade A to grade C, also without statistical significance.

Overall, these results showcase a pro-inflammatory profile linked with severe disease, marked by an escalation of pro-inflammatory markers, but not disease grade.

### 2.3. Pro-Inflammatory Cytokines Positively Correlate with Clinical Markers in PD Patients

Spearman’s correlation test was applied to the quantitative variables in this study to identify correlations between clinical measurements and the cytokine panel (Table 2). Initially, a significant positive correlation was found between IL-6 and the levels of CAL (*p* = 0.041). IL-1β exhibited significant positive correlations with all the measurements (*p* = 0.013 to PI, *p* = 0.01 to BOP, *p* = 0.002 to PPD, and *p* < 0.001 to CAL). The levels of IL-1RA inversely correlated with all measurements, however, without statistical significance. Consequently, a positive correlation was observed between clinical determinations and IL-1β/IL-1RA ratio, reaching statistical significance with BOP, PPD, and CAL levels (*p* = 0.020, *p* = 0.0015, and *p* = 0.022, respectively). No correlations were found between the levels of IL-23, IL-10, IFN-γ, TNF-α, and the clinical measurements. Interestingly, negative correlations were found between salivary IP-10 levels and BOP, PPD, and CAL (*p* = 0.045, *p* = 0.025 and *p* = 0.088, respectively). Overall, these results demonstrate correlations between the inflammatory profile and the phenotype of PD patients, as characterized by clinical measurements. 

## 3. Discussion

The inflammatory process during PD is described as complex and dependent on the bacterial and immunological status of the host. Periodontal bacteria trigger the host’s immune response, causing the release of inflammatory mediators and cytokines in the oral microenvironment [16]. Consequently, the pathogenesis of periodontitis leads to soft tissue destruction and bone resorption [9,17]. The immunological mediators detected in saliva, as well as in gingival crevicular fluid (GCF), are mostly pro-inflammatory cytokines, such as IL-1β, IL-6, and TNF-α, but anti-inflammatory and regulatory cytokines, such as IL-10 and IL1-RA, are also found to counterbalance the inflammatory response. In this process, the evolutionary factors of PD in terms of stage criteria seem to be relevant, as well as the type of cells present in the oral cavity and what they can secrete [17,18,19]. 

In a global perspective, our results provide a conceivable dual action of the immune response: one pro-inflammatory phase with the presence of IL-1β and IL-6, all along the disease development and mostly contributing to the increase of the disease severity rather than the rate of progression. This was also related to all clinical periodontal parameters (PI, BOP, PPD, CAL). The other phase involves an imbalance with the anti-inflammatory cytokines (IL-10 and IL-1RA) attempting to counteract the inflammatory immune condition. At present, the diagnosis of periodontitis is achieved using patient’s case history, clinical examination, and radiographic evaluation. Clinical parameters (PI, BOP, PPD, and CAL) are of great help to dentists; however, they only allow for perception after the onset of the disease. Our data illustrate and corroborate with other published studies [20,21,22], where clinic parameters are significantly higher in PD patients comparatively with HC, with a strong significant association with the disease severity (stage), but not with the risk of rapid progression (grade). Furthermore, the number of teeth was significantly decreased in PD patients with stage III/IV (*p* < 0.001) and in grade C (although not statistically significant) compared to HC. These findings align with other published works [13,23,24], also highlighting the relevance of tooth loss in PD progression. It is worth noting that tooth loss can be considered an independent indicator of an accumulation of oral inflammation [25] and a risk indicator factor of coronary heart disease in patients with PD [24].

Regarding the inflammatory factors, our data illustrate the predominance of pro-inflammatory cytokines in the development of PD, as evidenced by higher levels of IL-1β and IL-6 as disease becomes established, and consistent with previous studies [26,27]. The lower levels of IP-10 in PD patients suggest that its pro-inflammatory activity might be set aside. Alongside this pro-inflammatory trend, there is an anti-inflammatory response, marked by the effective presence of IL-10 and IL1-RA. These findings once again emphasize the importance of combining salivary biomarkers for the diagnosis of periodontitis. Several published studies confirm the utility of pairing IL-1β, IL-6, and MMP-8, achieving excellent sensitivity (≥83%) and specificity (≥81%), thereby enhancing overall diagnostic accuracy [21,28]. 

Numerous diseases’ susceptibility and severity are influenced by the balance between IL-1β and IL-1RA in local tissues [10,29,30]. As mentioned above, IL-1β, considered a key biomarker, is recognized as an important mediator in PD pathophysiology [9,26,31,32,33]. The IL-1 receptor antagonist, IL1-RA, regulates the local effect of IL-1β in inflammatory periodontal disease [34,35].

This was recently emphasized by the administration of IL-1RA-loaded dextran/PLGA microspheres, which significantly inhibited gingivitis and alveolar bone loss, offering the prospect of a new therapeutic target [30]. In our cohort, we observed an inverse relationship of IL-1β and IL1-RA cytokines. Higher levels of IL-1β and lower levels of IL1-RA were observed as PD developed. The work of Morelli (2014), which shows reduced IL-1RA levels in PD patients, supports our findings by indicating that salivary levels of IL-1RA and IL-6 may be useful markers for important changes in probing depth during gingival inflammation [36]. Consequently, higher IL-1β/IL-1RA ratios define a progressive worsening of PD, both in stage or grade situations, indicating the aggressiveness of the pro-inflammatory response and limited effectiveness of IL-1RA in mitigating the detrimental effects of IL-1β. Additionally, IL-1β/IL-1RA ratio was positively correlated and statistically significant with BOP, PPD, and CAL, supporting the notion that both IL-1β and IL-1RA are potential biomarkers or accurate predictors for diagnosing periodontitis, in agreement with the work of Wu et al., 2018 [37].

In this work, we extended the saliva cytokine analysis to other inflammatory (IL-6, IL-23, IFN-γ, TNF-α, and IP-10) and anti-inflammatory (IL-10 and IL1-RA) mediators. IL-6, a pro-inflammatory cytokine, is implicated in bone destruction during infection and has been found to be elevated in patients with periodontitis compared to healthy individuals [32,38]. Studies show that IL-6 levels increase with disease progression, particularly in severe cases, and are responsive to periodontal therapy [39]. The presence of IL-6 in saliva and GCF highlights its role in the development and progression of aggressive periodontitis. We observed that IL-6 levels were significantly higher in periodontitis stage III/IV compared to HC (*p* = 0.003) (Figure 3A), but also increased in Grade C, albeit without statistical significance (Figure 4A), validating, along with other studies, IL-6 importance [12,18]. Additionally, IL-6 levels were previously correlated with clinical indicators of periodontal health and are associated with a more aggressive disease phenotype [40]. Our data support that IL-6 is positively correlated with CAL (*p* = 0.041). In clinical practice, CAL is employed as a stage element in the severity dimension, offering a strong link between IL-6 and disease severity. 

TNF-α and IFN-γ are cytokines present, limiting the extent and duration of inflammatory processes [41]. For both cytokines, we did not find any statistical differences between the groups, whether in the stage or grade divisions, which corroborates previous studies [42]. 

CXCL10 (also known as IP-10) is an inflammatory cytokine, recruiting leukocytes and stimulating osteoclastogenesis [43,44] and has been implicated in various diseases, such as RA, autoimmune, and head/neck cancers [44,45,46]. CXCL10 was also reported to be present in higher levels in GCF and saliva, of PD patients compared to healthy controls [47,48]. Although these studies reported higher levels in PD patients, our cohort showed lower levels in PD regardless of stage or grade, possibly due to the modulation of periodontal disease by oral microbiota, including pathogenic bacteria, like *Poryphromonas gingivalis* and *Fusobacterium nucleatum* [49,50] which have been described to interfere with these inflammatory mediators [51,52]. 

IL-23, a pro-inflammatory cytokine produced by dendritic cells and monocytes/macrophages, is associated with the Th17 lineage and immune-related destructive tissue diseases [53]. The IL23/IL-17 axis has been linked to inflammation and exacerbated pathological events in various diseases [53,54]. While previous studies found elevated IL-23 levels in periodontitis [55,56], our study showed reduced IL-23 levels in PD patients, regardless of stage or grade classification. This contrasts with the existing research. We hypothesize that higher IL-23 levels may be associated with early disease development and later downregulated by IL-10, known to dampen the GCF IL-17 mediated inflammatory response [57].

The anti-inflammatory cytokine IL-10 is known to have a downregulating role [58]. Contradictory reports have been published regarding the role of IL-10 in PD. Some report higher salivary IL-10 in PD, while others report reduced or comparable IL-10 levels to healthy individuals [42,59]. In our study, we observed higher levels of IL-10 in stage III/IV periodontitis compared to stage I/II (*p* = 0.047) (Figure 3F), as well as in grade C periodontitis patients compared to grades A and B (although without statistical significance). Interestingly, Napimoga et al. (2011) also suggested a potential role for increased IL-10 levels in higher salivary IgA titers in chronic PD patients, in an attempt to control the inflammatory process [60]. In these conditions, the inflammatory process may be in its initial stages, where the presence of this anti-inflammatory cytokine inhibits part of the inflammatory network, or extrinsic factors present in their bacterial biofilm may favor the action of IL-10 [61]. In conclusion, the data on IL-23, IP-10, and IL-10 strongly support a kinetic evaluation of the inflammatory mediators throughout the development of PD.

From our data, we observed a classical pro-inflammatory profile with elevated levels of IL-1β and IL-6 in periodontitis patients. Even though IL-1RA was detected, it appeared insufficient to reverse the inflammatory process, as demonstrated by a higher IL-1/IL-1RA ratio in periodontitis subjects. In this group of Portuguese individuals, we found lower levels of IL-23 and IP-10 (CXCL10) in periodontitis patients but higher levels of IL-10. This may indicate a potential transition from an inflammatory stage toward homeostasis and resolution of the pathogenesis. The imbalance between IL-10 and IP-10 could reflect a shift in the oral bacteria profile, such as an altered ratio between *P. gingivalis* and *F. nucleatum* [49]. Our study reveals the complex network of immunomediators involved in the inflammatory process in PD. It also highlights that IL-1β and IL-6 are more useful to distinguish the disease severity than the clinical progression of the profile. Moreover, these cytokines show strong positive correlations with clinic parameters and hold promise as potential combined biomarkers for achieving a reliable diagnosis.

We acknowledge several limitations of our study as follows: the subdivision of groups diminished the statistical significancy of the study; there was no equal number of I/II and III/IV patients in our sample; other sociodemographic indicators could have been exploited, like, education level, income, occupation, life habits. In opposition, we highlight our strong points: we applied the current classification of periodontal diseases, the ability to analyze in a non-invasive matrix, and used the bead-based multiplex assay panel, which provides higher detection sensitivity and broader dynamic range. In our future approaches, to surpass the limitations of this study, we should enlarge the sample size, ensuring an almost identical number of patients in each stage and grade division. Additionally, we can evaluate this association of biomarkers using the GCF as a biological fluid to demonstrate that inflammatory mediators might locally support the severity process in PD. Furthermore, microbiome analysis would allow understanding of how bacterial interactions influence cytokine profiles.

## 4. Materials and Methods

### 4.1. Study Design and Ethical Approval

Throughout 12 months, between 2021 and 2022, convenience sampling was used to select patients from the Dental Clinic Unit of the University Institute of Health Sciences (IUCS-CESPU, Gandra, Portugal). The patients were then subclassified using the most recent consensus on periodontal disease classification from the American Academy of Periodontology/European Federation of Periodontology (AAP/EFP) [3]. Under reference CE/IUCS/CESPU-08/21, the study was submitted to and authorized by the IUCS ethics commission, and it was carried out in accordance with the Declaration of Helsinki. 

### 4.2. Study Population and Clinical Assessments

#### 4.2.1. Research Participants

The basis for determining the number of samples was performed assuming a non-parametric independent samples test and a medium effect size (0.4), a 5% significance level, a statistic of 80%, and equal sample sizes for each group. The total sample size calculated was 66 individuals, 22 for each group. However, no equal sample sizes for each group were obtained, since our sampling method was through convenience, based on patients that attended the appointments.

The goal and methods of the study were thoroughly explained to the patients both orally and in writing. Prior to the periodontal examination, the patients who agreed to participate in the study were required to sign an informed consent form and complete a questionnaire. The minimum number of natural teeth (at least 18) and the age range, between 18 to 70 years old, were the inclusion requirements. The exclusion criteria comprised the following: pregnant women; subjects with a current or previous history of oral and maxillofacial cancer, radiation or other mucosal pathology, having undergone periodontal treatment less than six months ago; individuals undertaking oncological treatment; those taking bone-related medication; individuals with a medical history of diabetes mellitus, hepatic or renal disease, or other serious medical conditions or transmittable diseases; individuals with a history of alcohol or drug abuse; those who had received antibiotic or any anti-inflammatory drug within the past 6 months; those with a routine use of oral antiseptics; individuals with implants or orthodontic appliances. Gender, age, smoking habits (smoking for at least a year), ex-smokers (quit smoking less than five years ago), non-smokers (has not smoked for more than five years), and oral hygiene practices (frequent brushing, use of dental floss, and interdental brushing) were among the recorded data from anamneses. The periodontal clinical data included the following: number of absent teeth; number of teeth with mobility; pocket depth (PPD), measured as distance from the gingival free-margin from the bottom of the pocket; gingival recession (REC) as the distance from the enamel–cement junction (CEJ) to the free gingival margin, (showing a negative signal whenever the gingival margin is located coronary at the CEJ); clinical attachment loss (CAL); plaque index (PI); bleeding on probing (BOP). These parameters were registered in six locations per tooth (mesio-vestibular, vestibular, disto-vestibular, mesio-lingual, lingual, and disto-lingual), using a CPITN 15 Hu-Friedy Europe Periodontal Probe, Rotterdam, the Netherlands. Full-mouth periapical radiographs were taken using long cone paralleling with Rinn holders. Wisdom teeth were excluded from the analysis. 

#### 4.2.2. Case Definition

The new AAP/EFP consensus [3] was used to classify periodontitis states. The following categories were applied to the periodontitis patient sites: (1) mild to initial to moderate periodontitis sites (CAL ≤ 4 mm and PPD ≤ 5 mm), and (2) severe to advanced sites (severe sites), with CAL and PD corresponding to those described in stages III and IV of periodontitis (CAL ≥ 5 mm) (3). For periodontitis diagnosis, stage I and II no tooth loss due to periodontitis; stage III tooth loss due to periodontitis ≤4 teeth; stage IV tooth loss due to periodontitis ≥5 teeth. As controls we used periodontal healthy individuals (healthy controls (HC)). The grade of periodontitis was evaluated considering the evidence or risk of rapid progression, in three categories: slow, moderate, and rapid progression denoted as Grade A, B, and C, respectively. The effects on patient’s systemic health were also considered as defined by Tonetti et al., 2018 [3]. Healthy sites from healthy people (PD ≤ 3 mm without BOP) [62] were also obtained and used as healthy controls (HC). 

### 4.3. Measurement Reliability and Reproducibility of Examiners

To ensure measurement accuracy, two senior periodontic specialists (referred to as FS and MR) underwent calibration. This calibration process involved 10 volunteers and occurred over two different days, with a 48 h gap between sessions. The calibration process consisted of both examiners independently measuring the same random group of volunteers, and the results were recorded to assess the level of reproducibility. The intra-examiner coefficients of correlation (CCI) for CAL and PD were 0.97 and 0.98 for both specialists, respectively. Additionally, the inter-examiner CCI values were 0.98 and 0.98 for both CAL and PD.

### 4.4. Collection of Salivary Samples

Unstimulated saliva samples were collected from each patient using the spitting method. Patients refrained from oral hygiene measures, eating, drinking, or chewing gum for at least 1h before sample collection. The samples were then stored at −80 °C for subsequent analysis [63]. 

### 4.5. Sample Preparation and Cytokine Quantification

Prior to assay, saliva samples were thawed and centrifuged at 6000× *g* for 10 min at 4 °C. The resulting supernatants were collected, and various aliquots were stored at −80 °C for further analysis. Cytokines levels in saliva were quantified in pg/mL, using the LEGENDplex™ Human Macrophage/Microglia Panel (13-plex) kit (Cat. 740503, BioLegend, San Diego, CA, USA), which allows quantification of the following immune mediators: IL-12p70, TNF-α, IL-6, IL-4, IL-10, IL-1β, arginase, CCL17 (TARC), IL-1RA, IL-12p40, IL-23, IFN-γ, and CXCL10 (IP-10). The assays were performed following the manufacturer’s instructions. Both the standard curve and the samples were acquired in an LSR II flow cytometer using the FACSDiva software (version 6.1.3) (BD Bioscience, Franklin Lakes, NJ, USA) and analyzed using the LEGENDplex v8 software (BioLegend, San Diego, CA, USA) [64]. For the cytokines included in this study, the detection limit was 2.44 pg/mL. The only exception was IL-1RA, whose detection limit was 122.07 pg/mL. Samples below detection limit were excluded from the analysis.

### 4.6. Statistical Analysis

Statistical analysis was performed using SPSS version 28 software (IBM, New York, NY, USA) and data were plotted using GraphPad Prism version 9 software (San Diego, CA, USA). 

Regarding the small sample size and the non-normality observed in our variables, the Kruskal–Wallis test was applied to identify statistical differences. For variables that attained global significance, pairwise comparisons were performed using Dunn’s multiple comparisons test. The chi-square test was performed for categorical variables to assess the dependence between variables. Correlations were measured using Spearman’s correlation coefficient.

## Figures and Tables

**Table 1:** Demographic characterization of the PD cohort.

	HC (n = 22)	Stage I/II (n = 17)	Stage III/IV (n = 29)	Statistical Analysis *	
				Effect Size	*p* Value
Gender, n (%)					
Male	3 (13.6)	8 (47.1)	10 (34.5)	0.26	0.070
Female	19 (86.4)	9 (52.9)	19 (65.5)		
Age, n (%)					
≤25 years	10 (90.9)	1 (9.1)	0 (0)	0.53	<0.001
≥26 years and ≤45 years	9 (42.9)	3 (14.3)	9 (42.9)		
≥46 years	3 (8.3)	13 (36.1)	20 (55.6)		
Smoking Habits, n (%)					
Smoker	3 (21.4)	1 (7.1)	10 (71.4)	0.32	0.049
Ex-Smoker	1 (12.5)	2 (25.0)	5 (62.5)		
Non-Smoker	18 (39.1)	14 (30.4)	14 (30.4)		

* For categorical variables chi-square test was used to access the dependence of variables; effect size measures (Phi or Cramer’s V to 2 × 2 comparisons or more, respectively) and *p* value are reported; n—number; %—percentage; *p*—level of significance.

**Table 2:** Correlation between periodontal clinical markers and cytokine.

	PI	BOP	PPD	CAL
IL-6 (pg/mL)	0.128	0.260	0.175	0.274 *
IL-1β (pg/mL)	0.299 *	0.381 **	0.368 **	0.395 **
IL-1RA (pg/mL)	−0.166	−0.121	−0.127	−0.096
IL-1β (pg/mL)/IL-1RA (pg/mL) Ratio	0.233	−0.295 *	0.307 *	0.290 *
IL-23 (pg/mL)	−0.199	−0.085	−0.138	−0.151
IL-10 (pg/mL)	0.186	0.285	0.175	0.248
IFNy (pg/mL)	0.236	0.247	0.195	0.215
TNFα (pg/mL)	−0.101	0.034	−0.048	−0.011
IP-10 (pg/mL)	−0.154	−0.274 *	−0.305 *	−0.235

* *p* < 0.05; ** *p* < 0.01 and data summarized as r of Spearman’s correlation coefficient. PI—plaque index; BOP—bleeding on probing; PPD—pocket depth; CAL—clinical attachment loss.

## Data Availability

The data can be accessed by contacting the corresponding author.
